# Definition of sinonasal and otological exacerbation in patients with primary ciliary dyskinesia: an expert consensus

**DOI:** 10.1183/23120541.00218-2024

**Published:** 2024-12-16

**Authors:** Myrofora Goutaki, Yin Ting Lam, Andreas Anagiotos, Miguel Armengot, Andrea Burgess, Raewyn Campbell, Mathilde Carlier, Nathalie Caversaccio, Neil K. Chadha, Berat Demir, Sinan Ahmed D. Dheyauldeen, Onder Gunaydin, Amanda Harris, Isolde Hayn, Deniz Inal-Ince, Eric Levi, Trini Lopez Fernandez, Jane S. Lucas, Bernard Maitre, Anne-Lise M.L. Poirrier, Lynne Schofield, Kazuhiko Takeuchi, Christine van Gogh, Nikolaus E. Wolter, Jean-François Papon

**Affiliations:** 1 Institute of Social and Preventive Medicine, University of Bern, Bern, Switzerland; 2 Paediatric Respiratory Medicine, Children's University Hospital of Bern, University of Bern, Bern, Switzerland; 3 Department of Otorhinolaryngology, Nicosia General Hospital, Nicosia, Cyprus; 4 Department of Otorhinolaryngology, and Primary Ciliary Dyskinesia Unit, La Fe University and Polytechnic Hospital, Valencia, Spain; 5 Medical School, Valencia University, Valencia, Spain; 6 Molecular, Cellular and Genomic Biomedicine Group, IIS La Fe, Valencia, Spain; 7 Primary Ciliary Dyskinesia Centre, Southampton Children's Hospital, Southampton NHS Foundation Trust, Southampton, UK; 8 Department of Otolaryngology, Royal Prince Alfred Hospital, Sydney, NSW, Australia; 9 Association ADCP, Saint-Étienne, France; 10 Department of Otorhinolaryngology, Head and Neck Surgery, University Hospital of Bern, University of Bern, Bern, Switzerland; 11 Division of Pediatric Otolaryngology-Head and Neck Surgery, Department of Surgery, BC Children's Hospital, University of British Columbia, Vancouver, BC, Canada; 12 Department of Otorhinolaryngology-Head and Neck Surgery, Pendik Training and Research Hospital, Marmara University Medical Faculty, Istanbul, Turkey; 13 Department of Otorhinolaryngology, Head and Neck Surgery, Oslo University Hospital, Oslo, Norway; 14 Faculty of Medicine, University of Oslo, Oslo, Norway; 15 Department of Otorhinolaryngology, Hacettepe University, School of Medicine, Ankara, Turkey; 16 Primary Ciliary Dyskinesia Centre, Southampton Children's Hospital, University Hospital of Southampton, Southampton, UK; 17 NIHR Respiratory Biomedical Research Centre, Clinical and Experimental Sciences, University of Southampton, Southampton, UK; 18 Department of Otorhinolaryngology, Head and Neck Surgery, Charité-Universitätsmedizin Berlin, Berlin, Germany; 19 Faculty of Physical Therapy and Rehabilitation, Hacettepe University, Ankara, Turkey; 20 Department of Otolaryngology, The Royal Children, Hospital Melbourne, Melbourne, VIC, Australia; 21 Asociación Española de Pacientes con Discinesia Ciliar Primaria, Santo Ángel, Spain; 22 Service de Pneumologie, Centre hospitalier intercommunal de Créteil, Unité de Pneumologie, GH Mondor, IMRB U 955, Equipe 8, Université Paris Est Créteil, Créteil, France; 23 Department of Otorhinolaryngology, University Hospital Liège, Liège, Belgium; 24 School of Medicine and Population Health, University of Sheffield, Sheffield, UK; 25 Paediatric Physiotherapy, Leeds Teaching Hospitals NHS Trust, Leeds, UK; 26 Department of Otorhinolaryngology-Head and Neck Surgery, Mie University Graduate School of Medicine, Tsu, Mie, Japan; 27 Department of Otorhinolaryngology-Head and Neck Surgery, Amsterdam UMC, Amsterdam, The Netherlands; 28 Department of Otolaryngology-Head and Neck Surgery, Hospital for Sick Children, University of Toronto, Toronto, ON, Canada; 29 Assistance Publique-Hôpitaux de Paris, Université Paris-Saclay, Hôpital Bicêtre, Service d'ORL, Le Kremlin-Bicêtre, France; 30 Faculté de Médecine, Université Paris-Saclay, Le Kremlin-Bicêtre, France

## Abstract

**Background:**

Recurrent infections of the nose, sinuses and ears are common problems for people with primary ciliary dyskinesia. While pulmonary exacerbations in primary ciliary dyskinesia are defined, there is no definition for ear-nose-throat exacerbations, a potential outcome for research and clinical trials.

**Methods:**

We set up an expert panel of 24 ear-nose-throat specialists, respiratory physicians, other healthcare professionals and patients to develop consensus definitions of sinonasal and otological exacerbations in children and adults with primary ciliary dyskinesia for research settings. We reviewed the literature and used a modified Delphi approach with four electronic surveys.

**Results:**

Definitions for both sinonasal and otological exacerbations are based on a combination of major and minor criteria, requiring three major or two major and at least two minor criteria each. Major criteria for a sinonasal exacerbation are 1) reported acute increase in nasal discharge or change in colour, 2) reported acute pain or sensitivity in the sinus regions and 3) mucopurulent discharge on examination. Minor criteria include reported symptoms, examination signs, doctor's decision to treat and improvement after at least 14 days. Major criteria for the otological exacerbation are 1) reported acute ear pain or sensitivity, 2) reported acute ear discharge, 3) ear discharge on examination and 4) signs of otitis media in otoscopy. Minor criteria are reported acute hearing problems, signs of acute complication, and doctor's decision to treat.

**Conclusion:**

These definitions might offer a useful outcome measure for primary ciliary dyskinesia research in different settings. They should be validated in future studies and trials together with other potential outcomes, to assess their usability.

## Shareable abstract

**These PCD-specific definitions of sinonasal and otological exacerbation might offer a useful outcome measure for research in different settings. Their usability needs to be validated in future studies together with other potential outcomes.** https://bit.ly/3XPLMpT

## Introduction

Dysfunction of motile cilia due to genetic mutations leads to a wide range of symptoms including multiple organ systems in patients with primary ciliary dyskinesia (PCD) [1, 2]. Despite the clinical heterogeneity, the greatest impact of impaired mucociliary clearance is seen on the respiratory tract and the ears [3]. Patients present with persistent wet cough and recurrent lower airway infections, progressing in time to irreversible lung damage [3]. Inadequate clearance of mucus, pathogens and debris in the nose and sinuses, as well as in the eustachian tube and middle ear, leads to bacteria growing in the mucus-clogged airways. Consequently, patients experience recurrent episodes of sinonasal infections, and the risk of sinonasal disease increases with age, with chronic rhinosinusitis (CRS) becoming a common feature as disease progresses [4–6]. With regards the ears, recurrent episodes of acute otitis media often progress to severe bilateral otitis media with effusion and conductive hearing impairment [7–11]. Acute infections of the nose, sinuses and ears in PCD usually involve already impaired upper airways, with a more complicated pathophysiology and course compared to common acute upper airway infections.

Respiratory exacerbations are a significant determinant of morbidity and subsequent care requirements of people with chronic respiratory diseases. They are typically characterised by deterioration of the patient's clinical condition, most often due to viral or bacterial infections or exposure to other triggering factors. Exacerbations often require additional management and have significant effects on disease progression, severity and patients’ quality of life [12–15]. For clinical and epidemiological research, exacerbations are important outcomes in measuring burden of disease or response to treatments [16, 17]. Despite their importance, there is a lack of evidence on the frequency of exacerbations, pulmonary, sinonasal and otological, among people with PCD, and their impact on quality of life has not been measured. The lack of evidence is partly explained by the lack of precise definitions. Pulmonary exacerbations have been defined, and were recently included in a core outcome set for pulmonary disease interventions [18, 19] in the framework of the Better Experimental Approaches to Treat Primary Ciliary Dyskinesia (BEAT-PCD) clinical research collaboration supported by the European Respiratory Society (ERS) [20, 21]. The existing definition excluded upper respiratory tract exacerbations because they often occur independently from lower respiratory tract exacerbations and have a different prognosis [18]. Therefore, despite their impact on the severity of PCD, there is still no definition for ear-nose-throat (ENT) exacerbations. This lack of definition is an important gap for future clinical research in the field, particularly because there are even fewer clinical outcomes capturing ENT disease in PCD than capturing lung disease [22].

Using an international panel of specialists involved in PCD care, we aimed to develop a consensus definition of ENT exacerbations for children and adults with PCD participating in clinical research.

## Methods

### Participants and purpose of the consensus

We established an expert panel consisting mainly of ENT specialists, with expertise in managing children and adults with PCD. Experts were invited to participate in the panel by the study coordinators. We invited specialists from PCD reference centres in Europe, particularly centres participating in the ENT Prospective International Cohort of PCD Patients (EPIC-PCD) study. We also contacted PCD reference centres internationally and requested contact details for ENT specialists with expertise in PCD who might be interested in joining the panel. We encouraged invited participants to suggest further members to ensure wide international representation, resulting in participating ENT specialists from Europe, Canada, Australia and Japan (supplementary table S1). Additionally, we invited a paediatric and an adult pulmonologist who were involved in the consensus group of the pulmonary exacerbations definition [18], and other healthcare professionals involved in PCD patient care and research. The panel was completed by two patient representatives, an adult with PCD and a parent of a child with PCD. The panel had 24 members in total, representing 13 countries. To ensure significant patient involvement and input from the people with first-hand experience of these exacerbations, we also set up a parallel group of patient and parent volunteers, with support from the European Lung Foundation [23], who did not join the consensus panel but provided feedback and were encouraged to participate in the surveys. The activities of the panel and the patient group were coordinated by two facilitators, a clinical epidemiologist with expertise in PCD research (MG) and a PCD PhD candidate (YTL); the latter did not participate in voting. An initial virtual panel meeting refined the aims and proposed methodology. The panel concluded that standardised definitions for PCD were missing and decided unanimously to produce two separate definitions: one for sinonasal exacerbations and one for otological exacerbations. Our goal was to establish definitions for use in research and clinical practice.

### Literature search

We conducted a systematic literature search of publications referring to ENT exacerbations in patients with PCD, or separately to sinonasal and otological exacerbations. Given the anticipated limited pre-existing literature on the topic, our search strategy was expanded at the outset to include other areas with common characteristics, in particular CRS. We searched PubMed for studies published between January 2012 and December 2021 using the following keywords: ciliary dyskinesia, primary OR immotile cilia syndrome OR Kartagener/ AND exacerbat* OR infect* OR acute/ AND sinus* for sinonasal exacerbations, or otit* OR ear or otol* for otological ones. We simplified the terms, excluding PCD-specific keywords, to expand on other diseases excluding the PCD-related keywords. We did not exclude any publication type or language.

### Reaching a consensus

A modified Delphi approach with online (eDelphi) surveys was used. Initial literature search results revealed that identified pre-existing definitions did not cover the need for PCD-specific definitions but could be used as a starting point for the first eDelphi survey. Based on these definitions and the panel consensus, we identified important components for definitions of sinonasal and otological exacerbations. For each survey, participants received detailed instructions and a link *via* email, then two reminders to respond within 2 and 3 weeks.

The panel decided that at least an 85% response rate would be required to proceed to the next survey and that 80% of agreement would signify consensus; however, the possibility of accepting a lower percentage agreement as consensus was left open provided that the panel were informed and there was no veto against it. Each survey (supplementary surveys) included different types of questions to reach consensus initially on the included components for each definition and subsequently on the details of specific components, *e.g.* elements of included components such as specific symptoms or signs. Each survey was designed based on the results of the previous survey and included a summary of these for the panel's information. Participants were asked to explain their opinions in free text boxes, particularly where consensus was not achievable despite high agreement, so statements could be clarified and modified in the next round. The number of surveys was not predefined, but ultimately four surveys were required. A virtual meeting was organised with MG and the patient group to explain details of the project to the patient and parent members and answer their questions. Replies remained anonymous to the panel and only the facilitators had access to identifying information. After the eDelphi surveys were completed, the results of the final survey were circulated among the panel to ensure all members agreed with the final definitions.

## Results

### Literature search

Our search resulted in 2352 abstracts related to sinonasal exacerbations and 2208 related to otological exacerbations. We did not identify any abstracts with definitions specific to PCD. After excluding duplicates and screening the abstracts, we identified 24 manuscripts that referred to sinonasal exacerbations. After searching their references, we identified six additional manuscripts (n=30 studies in total), including one systematic review [24]. A summary of definitions used in the literature for sinonasal exacerbation in patients with CRS in the identified studies is presented in table 1 [24–53]. These definitions were discussed at a virtual expert panel meeting and the elements they used were considered for developing the initial survey. No study fulfilled the criteria of otological exacerbation of a chronic condition.

**TABLE 1:** Summary of definitions used in literature for sinonasal exacerbation in patients with CRS

Definition	References
Acute increase in **severity** of sinus disease symptoms	[25]
Sudden **worsening** of CRS symptoms with a **return** to baseline symptoms, often after **treatment**	[26, 27]
Acute worsening of pre-existing CRS symptoms with **subsequent return to baseline** symptoms **with or without endoscopic evidence**	[26, 28]
Previous **diagnosis of CRS** exists, and a sudden worsening of symptoms occurs, with **a return to baseline symptoms following treatment**	[29–33]
Presence of **purulence on endoscopy** during a symptomatic exacerbation of CRS	[29, 34]
Sudden **worsening of pre-existing** CRS symptoms is called a CRS exacerbation	[35]
Diagnosis of chronic rhinosinusitis and acute exacerbation of CRS according to the criteria described in the European position paper on rhinosinusitis and nasal polyps	[36, 37]
Previous **diagnosis of CRS** but patient experiencing **acute** exacerbation **of symptoms**	[38–40]
**Acute** worsening of symptoms with return to baseline, often requiring a transient escalation in **treatment**, such as a course of oral antibiotics or corticosteroids	[39–42]
**Acute** exacerbation of CRS was defined as having received an a**ntibiotic** prescription **for worsening sinus symptoms**	[43]
Acute exacerbations among **surgically managed** CRS patients were defined as a post-endoscopic sinus surgery, after **90 days post-operation**	[44]
Acute bacterial CRS exacerbations (**patient-reported** sinus **infections** and CRS-related **antibiotic use**)	[45]
Sudden **worsening of baseline** symptoms (or developing **new symptoms**) in a patient with an established CRS diagnosis	[46]
Sudden worsening of the baseline CRS with either **worsening or new** symptoms; typically, the acute (not chronic) symptoms **resolve completely** between exacerbations	[47]
**Worsening**, with subsequent **resolution**, of symptoms in a patient carrying the **diagnosis of CRS**	[48]
Defined by minimum **SNOT-20** score of 1.0 on a scale of 0 to 5	[49]
Worsening of symptoms: facial **pain** or pressure, nasal **obstruction**, nasal **discharge**	[50,51]
Presence of **increased nasal congestion** and **facial pain**, **increased sinonasal discharge**, usually presence of an unscheduled sick visit (*i.e.* not a routine follow-up)	[24, 52]
**Acute** exacerbation of CRS was defined in a patient in whom a previous diagnosis of CRS exists, and a **sudden worsening of symptoms** occurs, with a **return to baseline** symptoms **following treatment**	[24, 29]
Natural exacerbation was defined as **patient-reported worsening of sinonasal symptoms** (*i.e.* runny nose, nasal congestion and nasal obstruction)	[24, 53]
History of **sudden worsening of pre-existing symptoms** suggests an acute exacerbation of CRS, which should be diagnosed by similar criteria and treated in a similar way to acute rhinosinusitis	[24, 40]
Self-reported **medication use** (antibiotics and oral corticosteroids) for **worsened nasal and sinus symptoms**, self-reported **worsened purulence** in the past 4 weeks	[24, 39]
Systemic **antibiotics**, systemic **corticosteroid**, plans for a **semi-urgent surgical intervention**, emergency department or **urgent care visit** or a **hospitalisation** for CRS	[24]
**Worse nasal symptoms**	[24]

CRS: chronic rhinosinusitis; SNOT-20: Sino-Nasal Outcome Test.

### eDelphi surveys

Response rates to the eDelphi surveys ranged between 88% and 100% (supplementary table S1). Two to five members of the patient group completed each survey. In survey 1, the panel assessed opinions about the importance of sinonasal and otological exacerbations for people with PCD and components that should be included in the exacerbation definitions. Consensus was reached that exacerbations from the nose and sinuses are an important problem for both adults and children with PCD; they impact the quality of life of people with PCD and can be an important outcome measure for ENT clinical trials in PCD. Opinions were similar for otological exacerbations; however, no consensus was reached on the importance of this problem for adults with PCD, primarily due to smaller frequency of acute ear exacerbations in adulthood. The panel also agreed that sinonasal, otological and pulmonary exacerbations may occur separately from each other, highlighting again the importance of separate definitions. Responses to key questions about the components of the two definitions are presented in supplementary table S2. The combination of new symptoms or worsening of baseline symptoms and of new clinical signs or changes in clinical examination was voted as the best combination of components to define both sinonasal (93%) and otological (97%) exacerbations. No consensus was reached about including the following components: 1) changes in imaging for sinonasal exacerbations, 2) decision of ENT specialist to treat (for both definitions) or 3) complete resolution of any changes and return to baseline (for both definitions).

Survey 2 included questions on specific elements, particularly symptoms (supplementary table S3) and signs (supplementary table S4), for the sinonasal and the otological exacerbation definitions. Agreement was reached for three symptoms and two signs for each definition in this round. Items that achieved 60–79% agreement in survey 2 were discussed again in survey 3. Tables 2 and 3 follow the process of reaching a consensus for the two definitions step by step from survey 2 to survey 4 and the levels of agreement until consensus was reached, or not. Survey 2 also clarified that sinus imaging should not be an absolute requirement for the definition of a sinonasal exacerbation, with the main reasoning that it should be restricted for baseline assessment and for complications, and that it entails too much radiation and offers little in case of acute exacerbations (85% agreement).

**TABLE 2:** Process of reaching consensus for the items included in the definition of a sinonasal exacerbation

	Agreement (%)			Included in the definition (% of agreement)
	Survey 2	Survey 3	Survey 4	
Patient-reported acute increase in nasal discharge or change in discharge colour	100	–	–	Major criterion (100)
Patient-reported acute pain or sensitivity in the sinus region (*i.e.* around the nose, eyes, on the cheeks or forehead)	85	–	–	Major criterion (83)
Patient-reported acute blocked nose or worsening in chronic feeling of blocked nose	92	–	–	Minor criterion (78)
Patient-reported acute decreased sense of smell	69	58	74	Minor criterion (74)
Reduced quality of life evaluated by any sinonasal-specific quality of life questionnaire	73	50	61	Not included
Mucopurulent nasal discharge at examination	100	–	–	Major criterion (87)
Increased mucus production or postnasal drip at examination	92	–	–	Minor criterion (70)
Signs of acute complication (*e.g.* orbital infection or abscess, meningitis, cerebral infection, cranial nerve palsy) at examination	72	52	83	Minor criterion (83)
Acute frontonasal or maxillary tenderness at examination	–	–	65	Not included
Doctor's decision to treat, not necessarily with antibiotics but also with increased upper airway clearance or other medication	–	81	–	Minor criterion (91)
Important improvement in symptoms reported by the patient or parent or in clinical findings in case further examination is possible, after a period of at least 14 days	–	80	–	Minor criterion (74)

Items that reached ≥80% were automatically included in the definition. Items that achieved 60–79% agreement in survey 2 were discussed again in survey 3. Items that achieved 50–79% agreement in survey 3 and newly suggested items by several members were discussed in survey 4. At survey 4, members voted whether items should be considered as major or minor criterion or be included at all. We considered reaching consensus at ≥80% agreement for major criteria and ≥74% for minor criteria; items with <74% agreement were not included at all.

**TABLE 3:** Process of reaching consensus for the items included in the definition of an otological exacerbation

	Agreement (%)			Included in the definition (% of agreement)
	Survey 2	Survey 3	Survey 4	
Patient-reported acute ear sensitivity or pain	92	–	–	Major criterion (91)
Patient-reported acute ear discharge	92	–	–	Major criterion (91)
Patient-reported acute hearing problems or worsening in pre-existing hearing problems	85	–	–	Minor criterion (74)
Reported feeling of fullness in the ears	77	58	57	Not included
Ear discharge at examination	92	–		Major criterion (83)
Signs of otitis media in otoscopy (*i.e.* erythema, collection)	92	–		Major criterion (87)
Signs of acute complication (mastoiditis, meningitis, cerebral abscess, facial or other cranial nerve palsy) at examination	69	46	78	Minor criterion (78)
Impaired hearing tested by pure-tone audiometry	69	62	70	Not included
Perforated eardrum at examination	62	54	43	Not included
Horizontal nystagmus at examination	35	–	14	Not included
Doctor's decision to treat, not necessarily with antibiotics but also with other medication	–	88	78	Minor criterion (78)
Important improvement in symptoms reported by the patient or parent or in clinical findings in case further examination is possible, after a period of 14 days	–	72	70	Not included

Items that reached ≥80% were automatically included in the definition. Items that achieved 60–79% agreement in survey 2 were discussed again in survey 3. Items that achieved 50–79% agreement in survey 3 and newly suggested items by several members were discussed in survey 4. At survey 4, members voted whether items should be considered as major or minor criterion or be included at all. We considered reaching consensus at ≥80% agreement for major criteria and ≥74% for minor criteria; items with <74% agreement were not included at all.

Survey 3 discussed elements from previous surveys, which had scored highly but not yet reached a consensus on inclusion (supplementary table S5). The panel unanimously agreed in this survey to introduce major and minor criteria for both definitions. This had been discussed as a possibility at the first panel meeting and was raised again at this point in the project, with the suggestion that the most important criteria be considered as major criteria and the rest as minor, according to the level of agreement reached for each. We also reached consensus (85%) that all clinical signs or changes seen in clinical examinations included in both definitions should be assessed in relation to previous examinations. In survey 4, participants voted specifically about major and minor criteria. For criteria for which consensus (≥80% agreement) was already reached, the panel was asked to vote whether they should be included as major or as minor criteria (tables 2 and 3). Criteria that reached >50% but <80% agreement in survey 3 were now voted upon for including as minor criteria or excluding from the definitions.

Based on discussions that clinical practice may differ substantially from research practices, particularly by the non-PCD ENT specialist, although we originally considered that the definitions would also cover clinical practice, the panel decided (100% agreement) to include the following clarification: “These definitions are aimed to be used in research settings, especially in clinical trials, to define a sinonasal or otological exacerbation in patients with PCD”. The panel also agreed that a) three major or b) two major and at least two minor criteria are needed to define a sinonasal or otological exacerbation (table 4). Panel members were invited to submit alternative proposals in case of disagreement; no other proposals were submitted. For sinonasal exacerbation, we reached consensus on three major criteria (reported acute increase in nasal discharge or change in discharge colour, reported acute pain or sensitivity in the sinus region, and mucopurulent nasal discharge at examination) and six minor criteria (reported acute blocked nose or worsening in chronic feeling of blocked nose, reported acute decreased sense of smell, increased mucus production or postnasal drip at examination, signs of acute complication at examination, doctor's decision to treat, and important improvement in symptoms or clinical findings after a period of at least 14 days). For an otological exacerbation, we reached consensus on four major criteria (reported acute ear sensitivity or pain, reported acute ear discharge, ear discharge at examination, and sign of otitis media in otoscopy) and three minor criteria (reported acute hearing problems/worsening in pre-existing hearing problems, signs of acute complications at examination, and doctor's decision to treat). Major criteria were decided on ≥80% consensus and minor on ≥74%, which was agreed by the panel (tables 2 and 3). Lastly, the panel highlighted that no criterion was an absolute requirement for either definition (table 4).

**TABLE 4:** Definitions of a sinonasal and an otological exacerbation for children and adults with PCD participating in clinical research

	Major criteria (based on ≥80% consensus)	Minor criteria (based on ≥74% consensus)
**Sinonasal exacerbation**		
**All three of the major criteria or two major and at least two minor criteria** are needed to define a sinonasal exacerbation for children and adults with PCD in clinical research settings	• Patient-reported acute increase in nasal discharge or change in discharge colour • Patient-reported acute pain or sensitivity in the sinus region (*i.e.* around the nose, eyes, on the cheeks or forehead) • Mucopurulent nasal discharge at examination	• Patient-reported acute blocked nose or worsening in chronic feeling of blocked nose • Patient-reported acute decreased sense of smell • Increased mucus production or postnasal drip at examination • Signs of acute complication (*e.g.* orbital infection or abscess, meningitis, cerebral infection, cranial nerve palsy) at examination • Doctor's decision to treat, not necessarily with antibiotics but also with increased upper airway clearance or other medication • Important improvement in symptoms reported by the patient or parent or in clinical findings in case further examination is possible, after a period of at least 14 days
**Otological exacerbation**		
**Three of the following major criteria or two major and at least two minor criteria** are needed to define an otological exacerbation for children and adults with PCD in clinical research settings	• Patient-reported acute ear sensitivity or pain • Patient-reported acute ear discharge • Ear discharge at examination • Signs of otitis media in otoscopy (*i.e.* erythema, collection)	• Patient-reported acute hearing problems/worsening in pre‑existing hearing problems • Signs of acute complication (mastoiditis, meningitis, cerebral abscess, facial or other cranial nerve palsy) at examination • Doctor's decision to treat, not necessarily with antibiotics but also with other medication

**These definitions are aimed to be used in research settings, especially in clinical trials, to define a sinonasal or otological exacerbation in patients with PCD. No individual criterion is considered an absolute requirement.** PCD: primary ciliary dyskinesia.

## Discussion

An international panel of ENT specialists, pulmonologists, healthcare professionals and people with PCD agreed on consensus definitions of sinonasal and otological exacerbations in children and adults with PCD to be used in research, especially in clinical trials. This effort followed a similar approach to that used to develop a consensus definition of pulmonary exacerbations in PCD [18]. Although upper and lower airway disease in PCD should be managed holistically and exacerbations often occur simultaneously, or progress to involve the whole airways, our panel agreed that exacerbations from the nose, the sinuses and the ears require separate definitions [54]. They can occur individually and have different characteristics. Both are an important problem in children with PCD whereas in adults sinonasal exacerbations remain a major issue but otological exacerbations are less common.

The main strengths of the study were the international and multidisciplinary nature of the panel, and the inclusion of patients and parents of children with PCD, together with the added group of patient volunteers. We performed a thorough systematic review of the literature, expanding our search to other conditions, such as other types of CRS, that have similarities with PCD. We retained a high panel response rate throughout the study. Although the panel considered originally developing definitions that could also be used in clinical practice, we agreed during the process that this would not be feasible. Clinical outcome measures for research, even simple ones such as those provided here, need to be very clearly defined, while in clinical practice an exacerbation might need to be diagnosed based only on reported symptoms, often without any examination, requiring a less precise definition.

Our panel thoroughly discussed whether existing definitions specifically for CRS exacerbation could also be used for children and adults with PCD, without the need to develop disease-specific definitions. We considered all available definitions (table 1), particularly the latest European position paper on rhinosinusitis and nasal polyps [36], which defined acute exacerbation of CRS as worsening of symptom intensity with return to baseline CRS symptom intensity, often after intervention with corticosteroids or antibiotics. We reached consensus that none of them fully covered the purpose of a PCD-specific definition, although they highlighted important components that we then discussed. Most of the available definitions were not precise enough to be used as outcome measures for clinical trials and refer to a deterioration in symptoms in general without listing specific symptoms. Considering that patients with PCD grow accustomed to their chronic symptoms and tend to underestimate them, we aimed to refer to specific symptoms. The panel members also agreed on the need to include in the definition PCD-specific signs seen at simple examination, which were not part of most existing definitions. We found no eligible definitions that could be used as a starting point for otological exacerbations.

Throughout the process, our panel highlighted the need to select elements that could be assessed easily in different settings and would not require complex ENT examination or a specialist with expertise in PCD to assess them. Most criteria refer to symptoms or signs that can be observed in simple clinical examination, the most complex assessment included being otoscopy. Panel members agreed that patients with PCD often underestimate their upper airway symptoms, which are nonspecific and to which they have grown accustomed, highlighting the need to also consider simple signs in the definitions [4, 7, 55, 56]. This was also shown in a recent study from EPIC-PCD that reported a lack of correlation between sinonasal and otological symptoms with objective measurements [57, 58].

Two components that required long discussions and voting rounds were doctor's decision to treat and the need for improvement of the symptoms and signs. In both definitions, decision to treat was included as a minor criterion because it could occur regardless of an exacerbation (*e.g.* detection of *Pseudomonas aeruginosa* in a routine nasal or ear sample). The panel clarified that treatment should refer to the need for not only antibiotics but also other medication or management practices such as upper airway clearance, *e.g.* start or increased frequency of saline irrigation. Return to baseline was a term that was not found agreeable to most panel members. Even though improvement in symptoms and signs, where follow-up examination is possible, was included as a minor criterion for the sinonasal definition, participants agreed that it is difficult to measure improvement because deterioration is partly expected due to the chronic nature of the disease. Particularly in the case of acute ear exacerbations, this was not considered possible, and it was excluded entirely from the definition.

This initiative was developed in the framework of the BEAT-PCD ERS clinical research collaboration (https://beat-pcd.squarespace.com) as part of our efforts to define and promote the use of reliable clinical outcome measures for PCD trial and clinical research [20]. The evidence base for PCD treatment is small, and there are no trials that have specifically assessed the management of upper airways. One of the top priorities related to PCD research that was recently identified by experts in the field was to identify the most suitable clinical and patient-reported outcomes focused on the upper and lower airways to be used as end-points. These new definitions were developed to address a gap in disease-specific and relevant outcome measures for the upper airways. Given that more trials are needed to improve care of PCD and new potential therapies are in the pipeline, these definitions might offer a useful outcome measure in different research settings. It is important to use and validate them in future studies and trials to assess their usability together with other potential outcomes.

## Data Availability

All data generated for this project have been made available in the manuscript display items or supplementary information.
